# Prognostic factors for maxillary sinus mucosal thickening following Le Fort I osteotomy: a retrospective analysis

**DOI:** 10.1186/s40902-019-0195-3

**Published:** 2019-03-11

**Authors:** Masashi Iwamoto, Miki Watanabe, Masae Yamamoto, Masato Narita, Takashi Kamio, Takashi Takaki, Takahiko Shibahara, Akira Katakura

**Affiliations:** 1 Department of Oral Pathobiological Science and Surgery, Tokyo Dental College, 2-9-18 Kandamisaki-cho, Chiyoda-ku, Tokyo, 101-0061 Japan; 2 Department of Oral and Maxillofacial Surgery, Tokyo Dental College, 2-9-18 Kandamisaki-cho, Chiyoda-ku, Tokyo, 101-0061 Japan

**Keywords:** Le Fort I osteotomy, Maxillary sinus mucosal thickening, Orthognathic surgery, Prognostic factors

## Abstract

**Background:**

Le Fort I osteotomy is one of the surgical procedures now routinely and safely performed. It is possible to move the maxilla in three dimensions, but it is necessary to separate the bones around the maxillary sinus. Therefore, with surgery, maxillary sinus mucosal thickening occurs. By knowing the changes in the sinus mucosa after surgery and the factors affecting it, it is possible to better predict the outcomes of surgery and contribute to safer surgery. In this study, thickening of maxillary sinus mucosa before and after surgery in Le Fort I osteotomy was evaluated using multidetector-row computed tomography (MDCT) images, and the changes in mucosal thickening and the related factors were examined.

**Methods:**

Using MDCT images, the maxillary sinus mucosa of 125 patients who had undergone Le Fort I osteotomy was retrospectively evaluated before surgery, 1 month after surgery, and 1 year after surgery. On the MDCT images, the maxillary sinus was judged as mucosal thickening and classified into three grades according to the proportion occupying the maxillary sinus. In the evaluation of factors related to mucosal thickening, the following eight factors were examined: sex, age, diagnosis, operating time, amount of postoperative bleeding, with/without bone graft, with/without multisegmental osteotomy, and with/without macrolide therapy after surgery.

**Results:**

The mean age at the time of surgery was 25.6 ± 8 years. Of all 125 patients, 66 had bilateral thickening, 19 had unilateral thickening, and 40 had no thickening. Factors that were significantly related to mucosal thickening were the operative time for the maxilla, bone grafts, and macrolide therapy after surgery.

**Conclusions:**

Operative time for the maxilla, bone grafts, and macrolide therapy after surgery were found to be related to mucosal thickening. In addition, MDCT scanning 1 month after surgery was considered to be appropriate for evaluation of maxillary sinus mucosal thickening.

## Background

In orthognathic surgery for patients with jaw deformity, Le Fort I osteotomy in combination with a mandibular osteotomy is one of the surgical procedures that is now routinely and safely performed at many facilities. In Le Fort I osteotomy, it is possible to move the maxilla in three dimensions, but it is necessary to separate the bones around the maxillary sinus. Therefore, following Le Fort I osteotomy, inflammatory changes in the maxillary sinus mucosa, so-called maxillary sinus mucosal thickening, occur. Inflammatory changes in the maxillary sinus mucosa can sometimes be a risk factor for infection. However, there has been no study of the sinus mucosa after surgery, and experience suggests that the changes appear to resolve. By knowing the changes in the sinus mucosa after surgery and the factors affecting them, it is possible to better predict the outcomes of surgery and contribute to safer surgery. In this study, the thickening of maxillary sinus mucosa before and after surgery in Le Fort I osteotomy was evaluated using multidetector row CT (MDCT) images, and the changes in mucosal thickening and related factors were examined.

## Methods

### Patients

This retrospective study followed the guidelines of the Helsinki Declaration. It involved 125 patients who had undergone Le Fort I osteotomy at Tokyo Dental College Chiba Hospital (present—Tokyo Dental College Chiba Dental Center) in the 4 years from January 2011 to December 2014. Patients who had a jaw deformity with cleft lip and palate syndrome and who had marked mucosal thickening and maxillary sinusitis on preoperative imaging examinations were excluded. The details of skeletal diagnosis and surgery were shown in Table 1.

**Table 1:** Summary of the patients who underwent Le Fort I osteotomy

Skeletal diagnosis of maxilla	Number of patients	Without asymmetry		With asymmetry		
		Protrusion	Retrusion	Protrusion	Retrusion	–
Maxillary movement		Setback	Advance	Setback	Advance	Horizontal and/or vertical*
Male (mean age, 25.8 ± 8 years)	32	8	16	0	4	4
Female (mean age, 25.6 ± 9 years)	93	29	39	6	7	12
Total	125	37	55	6	11	16

*Advance and setback are not done, only horizontal movement and/or vertical movement

### Grading of maxillary sinus mucosal thickening

**Fig. 1:**
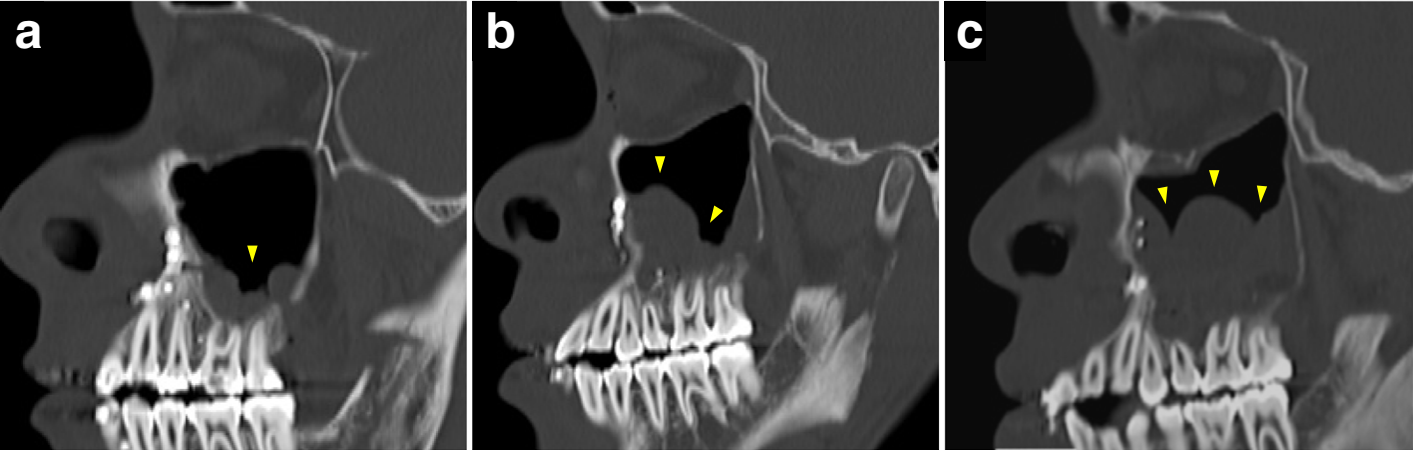
Grading of maxillary sinus mucosal thickening on MDCT images. **a** Grade 1, thickening of the maxillary sinus occupying the maxillary sinus volume is one third or less. **b** Grade 2, thickening occupying one third to two thirds of the maxillary sinus. **c** Grade 3, thickening occupying more than two thirds of the maxillary sinus

In all cases, imaging examinations of the head and neck (extraoral and intraoral radiography, MDCT examination, MRI examination) were performed before orthognathic surgery, and they were diagnosed by oral and maxillofacial radiologists. For all patients, MDCT scanning was performed with an MDCT scanner, SOMATOM Definition AS 64 (Siemens, Erlangen, Germany). Using MDCT images, the maxillary sinus mucosa of 125 patients was retrospectively evaluated before surgery, 1 month after surgery, and 1 year after surgery. On the MDCT images, a region showing a CT value (about 80 HU) similar to the soft tissue present in the maxillary sinus was judged as mucosal thickening and classified into three grades according to the proportion occupying the maxillary sinus (Fig. 1). Grading of maxillary sinus mucosal thickening was performed with reference to the evaluation method of the maxillary sinus mucosa by Yoshiura et al. [1], Carmeli et al. [2], and Bolger et al. [3].

The classification was as follows: grade 1, thickening of the maxillary sinus occupying the maxillary sinus volume is one third or less; grade 2, thickening occupying one third to two thirds of the maxillary sinus; and grade 3, thickening occupying more than two thirds of the maxillary sinus.

### Factors related to postoperative maxillary sinus mucosal thickening

In the evaluation of factors related to mucosal thickening, the following eight factors were examined: sex, age (mean age at the time of 1 month after surgery), diagnosis (skeletal diagnosis of the maxilla), operating time (total operating time and operating time at the end of Le Fort I osteotomy), amount of postoperative bleeding (total bleeding and bleeding at the end of Le Fort I osteotomy), with/without bone graft in Le Fort I osteotomy, with/without multisegmental osteotomy in Le Fort I osteotomy, and with/without macrolide therapy after surgery. The ultrasonic surgical method (piezoelectric surgery) was mainly used for the separation of the maxilla [4, 5]. In addition, macrolide therapy was implemented in accordance with previously reported research and guidelines [6–8].

### Ethical considerations

The postoperative MDCT examination including assessment of the maxillary sinus mucosa was thoroughly explained before the examination, and written consent was obtained from all patients. This study was approved by the Tokyo Dental College Institutional Review Board (Ethics Review Board Approval Number 803), and all participants provided their written, informed consent.

### Statistical analysis

Open source statistical software R version 3.2.3 was used for statistical analysis [9]. The factors were analyzed using paired *t* tests and chi-squared tests, as appropriate, comparing patients with and without mucosal thickening. *p* values < 0.05 were considered significant.

## Results

Table 1 shows the mean age and summary of male and female patients by diagnosis, by movement direction of the maxilla, divided by with/without asymmetry. There were three times as many females as males. Table 2 shows the characteristics of the patients in the two treatment groups with and without maxillary sinus mucosal thickening. Maxillary sinus mucosal thickening was observed in 85 (68%) patients on MDCT images at 1 month postoperatively. In 66 patients who showed bilateral mucosal thickening, 92 sinuses were grade 1, 31 were grade 2, and 9 were grade 3. In 19 patients with unilateral mucosal thickening, 14 sinuses were grade 1, 4 were grade 2, and 1 was grade 3 (Table 3). Of the 19 patients with unilateral mucosal thickening, 2 were asymmetrical, but no correlations were found between the 2 cases and the movement direction and movement amount of the maxilla. The details of the evaluations of factors related to mucosal thickening and the analysis results are shown in Table 4. There was a significant difference in operating time at the end of Le Fort I osteotomy, with/without bone graft, and with/without macrolide therapy.

**Table 2:** With/without maxillary sinus mucosal thickening on MDCT images 1 month after surgery

	Number of patients (*n* = 125)		
With maxillary sinus mucosal thickening	85	Bilateral	66
		Unilateral	19
Without maxillary sinus mucosal thickening	40

**Table 3:** Breakdown of maxillary sinus mucosal thickening in 125 patients 1 month after surgery

	Number of maxillary sinuses (*n* = 151)		
	Grade 1	Grade 2	Grade 3
Bilateral	92	31	9
Unilateral	14	4	1

**Table 4:** Details of the evaluation of factors affecting mucosal thickening and the analysis results at 1 month after surgery

			With mucosal thickening (*n* = 85)	Without mucosal thickening (*n* = 40)	*p* value
1)	Sex	Male	21	11	0.738
		Female	64	29	
2)	Mean age at the time of 1 month after surgery (years)		24.9 ± 7.9	27.1 ± 9.8	0.193
3)	Skeletal diagnosis of maxilla	Protrusion	27	10	0.323
		Retrusion	40	15	
		Protrusion with asymmetry	3	3	
		Retrusion with asymmetry	5	6	
		Asymmetry	10	6	
4)	Operating time (min)	Total operating time	375.3 ± 76.7	368.2 ± 80.7	0.638
		At the end of Le Fort I osteotomy	142.7 ± 47.4	117.5 ± 50.1	0.008*
5)	Bleeding (mL)	Amount of postoperative bleeding	310.4 ± 214.5	297.8 ± 229.0	0.765
		Amount of bleeding at the end of Le Fort I osteotomy	130.3 ± 112.3	124.3 ± 125.2	0.790
6)	Bone graft	With bone graft	29	22	0.027*
		Without bone graft	56	18	
7)	Type of osteotomy	One-piece Le Fort I osteotomy	69	36	0.209
		Multisegment Le Fort I osteotomy	16	4	
8)	Macrolide therapy	With macrolide therapy	19	3	0.031*
		Without macrolide therapy	66	37	

^*^p < 0.05 (paired *t* tests and chi-squared tests)

## Discussion

Since the application of Le Fort I osteotomy to orthognathic surgery in 1927 by Wassmund [10], improvements were made by many surgeons, and it became a surgical operation that was mostly established by Obwegeser [11]. Today, it is a procedure that is frequently performed due to its diversity of movement directions.

In Le Fort I osteotomy, the bone around the maxillary sinus is separated. Therefore, after surgery, blood accumulates in the sinus, and inflammatory changes (sinus mucosal thickening, edematous swelling) occur. It is also thought that the blood is absorbed with the progress of time, and the thickening of the sinus mucosa also disappears. It is easy to imagine that the risk of onset of maxillary sinusitis will be high if the blood reservoir or thickening of the mucosa persists for a long time. In the past, maxillary sinusitis has occurred, but fortunately, in the present evaluation period, there were no cases of maxillary sinusitis. Although mucosal thickening was not observed on the MDCT images of Le Fort I osteotomy after 1 year, mucosal thickening was observed in 68% in the first month after operation. Due to the characteristics of surgery, maxillary sinusitis may occur. Many reports on events after Le Fort I osteotomy are mostly related to abnormal fractures and bleeding [12–15]. However, to the best of our knowledge, there are very few reports on the incidence of maxillary sinusitis after Le Fort I osteotomy. Although Panula et al. [16] reported it in 6 of 655 patients, Kramer et al. [17] reported it in 11 of 1000 patients and Chow et al. [18] reported it in 3 of 125 patients; thus, the incidence and factors related to postoperative maxillary sinusitis have not yet been clarified.

In the present results, correlations with mucosal thickening were suggested, and factors that showed a significant difference were the operative time for the maxilla, bone grafts, and macrolide therapy after surgery. In 40 patients, no mucosal thickening was shown. One of the reasons for this may be that there was less blood retention in the maxillary sinus after surgery.

Due to the significant difference in operating time during Le Fort I osteotomy, it was suggested that a safer and faster procedure leads to the prevention of mucosal thickening. We believe that using the ultrasonic surgical method (piezoelectric surgery) and preoperative simulation by patient-specific 3D models made with a 3D printer contribute to safer and faster surgery [19]. Depending on the amount of movement of the maxilla, gaps between the bones may increase. Bone grafting promotes the formation of surrounding bone, and consequently, it was thought to contribute to the reduction of mucosal thickening. Furthermore, the results of this study support the role of postoperative macrolide therapy in reducing mucosal thickening. Opinions are divided on the timing of postoperative MDCT scanning to evaluate the maxillary sinus mucosa. Frequent MDCT scanning for the purpose of observation should not be done, since X-ray exposure should be avoided. Therefore, it is necessary to establish the validity of performing MDCT imaging. Currently, MDCT scanning is performed 1 month after surgery in our practice. Although it aims mainly to evaluate the condition of the bone, it also evaluates inflammatory changes of the maxillary sinus mucosa at the same time. If maxillary sinus mucosal thickening has been prolonged at that time, it is thought that the risk of infection remains high, and macrolide therapy is continued. Three-dimensional evaluation of the maxillary sinus by MDCT scanning is not performed to track the inflammatory changes of the maxillary sinus mucosa after scanning at 1 month postoperatively. If CT is performed, it is a two-dimensional evaluation such as Waters’ view. Therefore, it is difficult to demonstrate the dynamics over time. Therefore, while the period of continuing macrolide therapy is empirical, it is about 1 to 2 months. It is natural to observe the patient’s status carefully after surgery. In addition, it was demonstrated that MDCT 1 month after surgery looking for maxillary sinusitis can be helpful for deciding whether to continue postoperative macrolide therapy.

## Conclusions

In observation of maxillary sinus mucosal thickening using preoperative and postoperative MDCT images, shortening of the operative time, bone grafting, and macrolide therapy contributed to the prevention and reduction of mucosal hypertrophy following Le Fort I osteotomy. In addition, the usefulness of MDCT 1 month after surgery for determining whether to continue macrolide therapy was shown.

## Abbreviations

HU: Hounsfield units
MDCT: Multidetector-row computed tomography
MRI: Magnetic resonance imaging

## References

[1] Bolger WE, Woodruff WW, Morehead J, Parsons DS (1990). Maxillary sinus hypoplasia: classification and description of associated uncinate process hypoplasia. Otolaryngol Head Neck Surg.

[2] Yoshiura K, Ban S, Hijiya T, Yuasa K, Miwa K, Ariji E (1993). Analysis of maxillary sinusitis using computed tomography. Dentomaxillofac Radiol.

[3] Carmeli G, Artzi Z, Kozlovsky A, Segev Y, Landsberg R (2011). Antral computerized tomography pre-operative evaluation: relationship between mucosal thickening and maxillary sinus function. Clin Oral Impl Res.

[4] Takaki T, Ootake Y, Kogou T, Hirota M, Yamamoto M (2014). Use of ultrasonic new shape blade in orthognathic surgery: review of 138 patients. J Oral Maxillofac Surg.

[5] Matsuda H, Furuya Y, Sasaki H, Takanashi T, Morioka T (2015). Comparison of surface morphology and healing in rat calvaria bone defects between ultrasonic surgical method and rotary cutting method. J Hard Tissue Biol.

[6] Sasaki J, Kaneko A, Karakida K, Shiiki K, Sakamoto H (1995). Comparative clinical study of azithromycin with tosufloxacin tosilate in the treatment of acute odontogenic infection. Jpn J Antibiot.

[7] Shinkai M, Henke MO, Rubin BK (2008). Macrolide antibiotics as immunomodulatory medications: proposed mechanisms of action. Pharmac Therapeutics.

[8] Mandal R, Patel N, Ferguson BJ (2012). Role of antibiotics in sinusitis. Curr Opin Infect Dis.

[9] R Core Team (2018). R: a language and environment for statistical computing.

[10] Drommer RB (1986). The history of the “Le Fort I osteotomy”. J Oral Maxillofac Surg.

[11] Obwegeser H (1969). Surgical correction of small or retrodisplaced maxillae. Plast Recon Surg.

[12] Lanigan DT, West RA (1984). Management of postoperative hemorrhage following the Le Fort I maxillary osteotomy. J Oral Maxillofac Surg.

[13] Kim SG, Park SS (2007). Incidence of complications and problems related to orthognathic surgery. J Oral Maxillofac Surg.

[14] O’Regan B, Bharadwaj G (2007). Prospective study of the incidence of serious posterior maxillary haemorrhage during a tuberosity osteotomy in low level Le Fort I operations. Br J Oral Maxillofac Surg.

[15] Bhaskaran AA, Courtney DJ, Anand P, Harding SA (2010). A complication of Le Fort I osteotomy. Int J Oral Maxillofac Surg.

[16] Panula K, Finne K, Oikarinen K (2001). Incidence of complications and problems related to orthognathic surgery: a review of 655 patients. J Oral Maxillofac Surg.

[17] Kramer FJ, Baethge C, Swennen G, Teltzrow T, Schulze A (2004). Intra-and perioperative complications of the Le Fort I osteotomy: a prospective evaluation of 1000 patients. J Craniofac Surg.

[18] Chow LK, Singh B, Chiu WK, Samman N (2007). Prevalence of postoperative complications after orthognathic surgery: a 15-year review. J Oral Maxillofac Surg.

[19] Kamio T, Hayashi K, Onda T, Takaki T, Shibahara T (2018). Utilizing a low-cost desktop 3D printer to develop a “one-stop 3D printing lab” for oral and maxillofacial surgery and dentistry fields. 3D Print Med.

